# Schools caught in conflict: lessons from Honduras on violence, vulnerability, and educational barriers

**DOI:** 10.3389/fpsyg.2025.1689812

**Published:** 2025-12-03

**Authors:** Miguel Landa-Blanco

**Affiliations:** School of Psychological Sciences, Faculty of Social Sciences, National Autonomous University of Honduras, Tegucigalpa, Honduras

**Keywords:** violence, vulnerability, educational barriers, conflict, schools, low and middle income countries (LMIC), violence prevention, structural violence

## 1 Introduction

Schools can help prevent violence by engaging children in education and organized activities, supporting positive parenting, and challenging harmful social norms such as gender-based violence. Skilled teachers and school programs also provide early opportunities to influence young people and reduce the risk of future aggression (World Health Organization, 2019). What, then, occurs when schools themselves are embedded in contexts of structural violence that endanger both teachers and students? Structural violence refers to the indirect harm embedded within social, political, and economic systems that obstruct individuals or groups from meeting basic needs or achieving their full potential. It manifests through unequal access to resources, power, and opportunities, resulting in preventable suffering without an identifiable perpetrator (Burton et al., 2021).

Structural violence within educational settings occurs through institutional policies and practices that marginalize certain student groups and obstruct equitable access to learning. When schools operate within contexts of structural violence, they often reproduce the same inequities and harms they are meant to prevent. Structural factors such as political instability and community violence intersect within the school environment, reinforcing cycles of exclusion and fear. As a result, the school's protective role erodes, and the very systems designed to safeguard children instead deepen their exposure to harm (Brissett et al., 2025). Evidence from low- and middle-income countries (LMICs) reveals that both girls and boys are regularly exposed to various forms of violence in and around schools, often inflicted by fellow students, teachers, or other members of the community (Evans et al., 2025).

**Figure 1:**
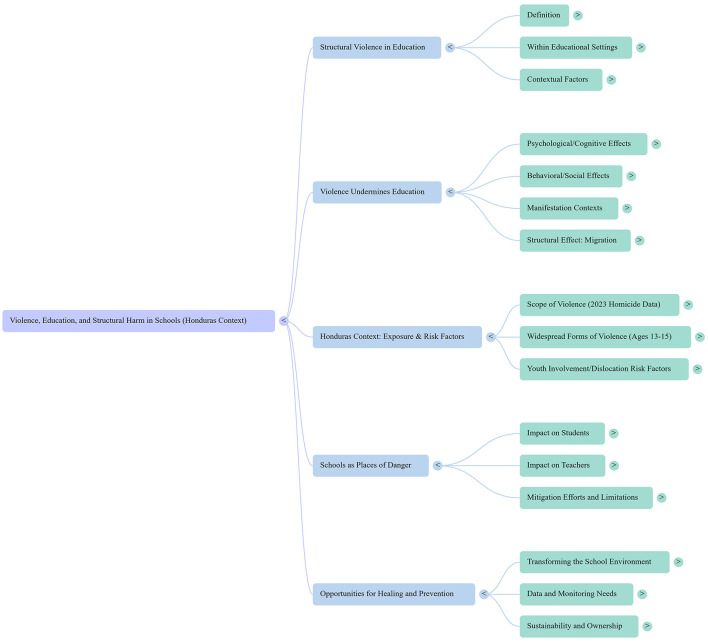
Manuscript overview. This diagram was generated using Google Notebook's LM mind map feature.

This opinion manuscript provides an overview of the Honduran setting, examining how schools are entangled in broader dynamics of violence that create risks for both teachers and students. By drawing on recent international academic studies, as well as national and institutional reports, it argues that schools in Honduras are not only potential sites for violence prevention but also spaces where structural violence, vulnerability, and educational barriers converge. The central thesis is that understanding schools as both victims and potential agents of change is essential for designing policies that safeguard education and address the broader conditions fueling violence. While grounded in the Honduran context, these lessons may also be applied to other countries facing similar intersections of conflict, inequality, and fragile educational systems. Figure 1 presents a mind map of the content included in this manuscript.

## 2 The many ways violence undermines education

This section examines how exposure to violence disrupts children's educational trajectories through psychological, behavioral, social, and structural mechanisms. By tracing these interconnected effects, it highlights how schools in violent contexts become both sites of vulnerability and critical spaces for potential recovery.

In this sense, violence affects children's education in direct and multifaceted ways. Exposure to violence can cause children to avoid attending school (Rankine et al., 2022). For those who do attend, the psychological toll of chronic exposure to trauma manifests in difficulties with concentration, memory, and impulse control; cognitive functions that are critical for learning.

Empirical evidence emphasizes the magnitude of these effects. Results from a meta-analysis demonstrate that exposure to any form of violence during childhood is significantly associated with poorer educational outcomes. Specifically, children who have experienced violence face a 13% increased likelihood of not completing their education. Male students who are victims of bullying are nearly three times more likely to be absent from school. In contrast, female students who have endured sexual violence exhibit a similarly elevated risk of school absenteeism. Furthermore, experiences of childhood violence are linked to reduced academic performance on standardized assessments, evidencing the detrimental impact on cognitive and educational development (Fry et al., 2018).

The psychological consequences of violence extend beyond academic performance. For example, experiencing high levels of violence during pre-adolescence can result in emotional numbing and the development of externalizing behaviors (Mrug et al., 2016). Such behaviors frequently lead to disciplinary actions rather than supportive interventions, driving vulnerable children further away from educational opportunities. Complementary, childhood exposure to violence also leads to a higher risk of suffering internalizing problems (Hardaway et al., 2012), which, in turn, are associated with lower academic achievement (Pedersen et al., 2019).

Additionally, exposure to violence across different contexts (home, school, and community) has distinct and compounding effects on children's and adolescents' mental health and behavior. Studies have revealed that witnessing violence at school was linked to increased anxiety and depression; at home, it predicted both anxiety and aggression; and in the community, it was associated with later delinquency. Victimization at home contributed to anxiety, depression, and aggression, while victimization at school was explicitly related to anxiety (Mrug and Windle, 2010). These results directly reinforce the manuscript's central argument. In contexts such as Honduras, where children face overlapping forms of violence, schools operate not only as sites of learning but also as environments shaped by risk and vulnerability.

Moreover, violence disrupts the social environment of schools. Students may struggle to form secure attachments (Fleckman et al., 2022), leading to isolation, reduced participation, and lower school functioning (Koposov et al., 2021). This erosion of social bonds undermines the school's potential as a nurturing community, increasing dropout rates and limiting prospects. In Honduras, where resources are limited and violence is widespread, the education system struggles to meet the complex needs of traumatized children, with profound implications for their life trajectories.

Finally, violence in the Northern Triangle (Honduras, Guatemala, and El Salvador) significantly drives unaccompanied child migration to the United States. This link remains strong even after controlling for economic factors and policy changes, highlighting violence as a primary force behind migration from the region (Clemens, 2021). This migration often interrupts children's education, disrupting their development and future opportunities.

## 3 Violence shapes the lives of children in Honduras

This section explores how widespread violence in Honduras affects children's development, socialization, and access to education. It highlights the multiple environments through which violence shapes children's daily lives within the school and community settings. In this sense, violence in Honduras is woven into the everyday experiences of many children, shaping their development in profound ways.

Homicide data from 2023 indicates that 170 victims were under the age of 18, accounting for 5.06% of the total reported homicides across the country (IUDPAS, 2024). However, violence against children is not limited to the streets or public spaces; physical, emotional, and sexual abuse occur with alarming frequency (Landa-Blanco et al., 2024, 2025). These early experiences of violence profoundly affect a child's sense of safety and trust (Brown, 2023).

Beyond the home, many communities are controlled or heavily influenced by gangs that expose children and adolescents to constant threats, coercion, and sometimes forced recruitment, forcing them to navigate a fractured social fabric where protective institutions often fail (McGrath, 2024). Schools occupy a complex and contradictory role within this environment: while they hold the potential to provide refuge and opportunity, they frequently mirror the violence of their communities. Thus, they become sites where bullying, intimidation, and neglect are common, complicating efforts to use education as a pathway out of cycles of violence and poverty. A national study examining violence among Honduran students aged 13–15 attending public schools surveyed 943 participants. The findings reveal widespread exposure to multiple forms of violence (Suazo and Cruz, 2022):

- Domestic verbal abuse: 34.2%
- Domestic physical maltreatment: 24.7%
- Peer violence- physical aggression: 43.3%
- Bullying: 38.2%
- Verbal mistreatment from peers or siblings: 37.4%
- Property damage: 50.4%
- Physical attacks without weapons: 49%
- Robbery by force: 43.2%
- Sexual harassment: 13.8%
- Severe sexual violence: 5%−8%
- Witnessed street fights: 32.3%
- Observed thefts or violent incidents at home: 30%

Complementarily, a qualitative study of underage Honduran offenders found that several critical risk factors contribute to their involvement in illegal activities and hinder their development. These include parental neglect, family separation, and having relatives engaged in criminal behavior. Many youths spend extended periods away from home or leave entirely, often starting work early, which restricts their educational opportunities (IDLO and INAMI, 2021). Taken together, these data illustrate the interaction of violence, poverty, and social dislocation in shaping children's lives.

## 4 Schools as places of danger

This section examines how community violence penetrates educational spaces in Honduras, shaping the daily realities of both students and teachers. Although schools ideally serve as safe spaces for children, in many Honduran communities, they are far from immune to the surrounding violence. A qualitative study conducted with children involved in the justice system as offenders revealed that many schools are heavily influenced by gangs operating both within and around school grounds. These gangs use intimidation and coercion to recruit students and, in some cases, force them to participate in drug trafficking. Observational visits to selected schools confirmed the presence of gang-related graffiti inside classrooms, evidencing the pervasive infiltration of gang activity within the educational environment. Such conditions contribute to high rates of absenteeism and school dropout, further exacerbated by unsafe environments characterized by gang presence, extortion, and drug trafficking and consumption (IDLO and INAMI, 2021).

Importantly, the reach of violence within schools extends beyond students to educators. A national survey found that 46% of teachers had experienced violence in or near their schools. The most common incidents involved robberies, assaults, sexual harassment, and extortion, with less frequent but severe cases of attempted homicide and kidnapping. In addition, 76% of teachers reported receiving direct threats to their lives, freedom, or physical safety, most often from parents, students, or gangs. Altogether, more than 300 teachers reported being forcibly displaced within the country as a result of violence (Comité Nacional de Docentes et al., 2024). Without measures that protect both students and teachers, the role of schools as spaces of learning and development is severely undermined.

These figures reveal the invasive nature of violence across the social ecology of children's lives. For many, school is not an escape from violence but another arena in which it unfolds. Recognizing this dual reality is critical: schools are themselves victims of the broader violent context, yet they remain among the few institutions capable of mitigating its effects through prevention, support, and resilience-building (Brissett et al., 2025).

In this line, the Honduran police have implemented interventions through the “School Police,” a community-oriented approach that engages directly with students and school staff. Programs such as Gang Resistance Education and Training (GREAT) provide targeted support in prioritized schools, focusing on the promotion of values, conflict resolution, and the prevention of violence, alcohol, and drug use. These initiatives aim to create safer educational environments, foster positive relationships between law enforcement and students, and mitigate the influence of gangs and criminal activity within schools (Abate-Flores et al., 2020).

However, research suggests that GREAT may overlook the broader political and social dynamics shaping school violence. Teachers and students often perceive a disconnect between the program's goals and the realities of gang influence (McGrath, 2024). While GREAT represents a proactive step toward violence prevention, its effectiveness may be limited unless interventions are attuned to local contexts, political realities, and the experiences of those affected by violence.

## 5 Discussion

Despite these challenges, schools hold extraordinary potential as sites of healing and prevention if they are supported and restructured accordingly. Because schools are often the only consistent institutional presence in children's lives, they offer a critical touchpoint for early identification of trauma and timely intervention. Transforming schools into trauma-responsive environments will require concerted action across multiple sectors. The Ministries of Education and Health must collaborate closely to institutionalize mental health services within the school system. Teacher training programs should incorporate extensive education on trauma, violence prevention, and trauma-sensitive classroom management techniques (Koslouski et al., 2023). At the same time, standardized protocols for identifying, reporting, and responding to abuse need to be established. Equally important is the formal integration of civil society organizations with expertise in child protection and psychosocial support into the school ecosystem.

Reliable data and monitoring are equally essential to inform these efforts. In this line, a global review of school-related violence data evidences a critical gap in the capacity of LMICs to monitor, prevent, and respond to violence within educational settings (Evans et al., 2025). In this sense, several LMICs lack basic statistics to guide effective interventions. For instance, only about one in six countries have data on recent sexual violence perpetrated by school staff, an indicator critical for shaping protective policies. This scarcity of data is especially acute for younger children, making early detection and response nearly impossible. In Honduras and comparable contexts, this absence of reliable monitoring creates a dual vulnerability: children continue to face exposure to violence in and around schools, while weak institutional data systems hinder accountability and evidence-based policymaking.

The historical reliance on external assistance further compounds this challenge. For years, international initiatives helped fill Honduras's institutional gaps in data collection and violence prevention. USAID previously supported key programs in Honduras that reduced youth violence and promoted safer schools. For example, *Asegurando la Educación* fostered nonviolence, trained teachers, and built early-warning systems (DAI Global, 2023), while *Proponte Más* worked with 800 high-risk families to lower delinquency, substance abuse, and antisocial behavior, and to support youth reintegration through family counseling (Creative Associates International, 2016). Their abrupt termination left a significant gap in violence-prevention efforts, removing crucial technical and financial resources. It also resulted in the suspension of research, the loss of valuable information, and the decline of institutional knowledge (Elvevåg, 2025). This disruption urges Honduras to take greater ownership of violence prevention, integrate trauma-informed and restorative practices into education and health systems, and build national mechanisms to monitor and respond to school-based violence. With coordination among ministries, local governments, and civil society, Honduras can move from external aid dependence to a locally sustainable framework that tackles the structural roots of violence through community empowerment.

Specifically, the national government can capitalize on the pool of professionals who formerly worked for USAID-funded programs by integrating them into public institutions and community initiatives. These individuals possess specialized expertise in violence prevention, education management, psychosocial support, and monitoring and evaluation. Such skills are essential for sustaining progress in the absence of external funding. By redeploying this workforce through the Ministries of Education, Security, and Social Development, the government can strengthen intersectoral coordination and ensure continuity in school and community safety interventions. Additionally, creating national technical units or advisory councils that draw on these professionals' experience could help institutionalize evidence-based practices and expand trauma-informed programming. Leveraging this existing human capital would mitigate the loss of USAID's institutional capacity and advance Honduras's goal of sustainable systems for preventing violence.

In conclusion, addressing the impact of interpersonal violence on education is not merely about improving academic outcomes. It is about recognizing and responding to the deep trauma that undermines children's ability to learn, grow, and thrive. Schools have the unique capacity to be places where violence is detected early, interrupted, and transformed into opportunities for healing and resilience (World Health Organization, 2019). For Honduras to break the cycle of violence that traps so many of its children, education must become a vehicle for both knowledge and recovery. This will require shifting our perspective to see schools not just as sites of instruction, but as critical environments for restoring dignity and safety to the country's most vulnerable young people (Brissett et al., 2025). However, this opinion article is limited by its reliance on secondary data and the potential underreporting present in national statistics. Future studies should prioritize the generation of primary, context-specific data to more accurately capture the scope and nuances of interpersonal violence and its educational consequences. These include longitudinal research to understand long-term effects, as well as participatory approaches that incorporate the voices of children, teachers, and communities. Such evidence is urgently needed to inform effective policies and interventions that can genuinely disrupt the cycle of violence and foster safer, more supportive learning environments.

## References

[B1] Abate-Flores C. E. Urtecho-Osorto Ó. Landa-Blanco M. Ávila Flores J. Reyes Flores L. (2020). Implicaciones teóricas y prácticas de la adopción del Modelo de Servicio de Policía Comunitaria en Honduras. Rev. Logos Cienc. Tecnol. 12, 85–96. doi: 10.22335/rlct.v12i2.1137

[B2] Brissett D. Rankine J. Mihaly L. Barral R. Svetaz M. V. Culyba A. . (2025). Addressing structural violence in school policies: a call to protect children's safety and well-being. J. Adolesc. Health 76, 752–756. doi: 10.1016/j.jadohealth.2025.01.027 40117388 PMC12150906

[B3] Brown D. (2023). Childhood experiences, growing up “in care,” and trust: a quantitative analysis. Child. Youth Serv. Rev. 144:106734. doi: 10.1016/j.childyouth.2022.106734

[B4] Burton C. W. Gilpin C. E. Draughon Moret J. (2021). Structural violence: a concept analysis to inform nursing science and practice. Nurs. Forum 56, 382–388. doi: 10.1111/nuf.12535 33355920

[B5] Clemens M. A. (2021). Violence, development, and migration waves: evidence from Central American child migrant apprehensions. J. Urban Econ. 124:103355. doi: 10.1016/j.jue.2021.103355

[B6] Comité Nacional de Docentes Save the Children, and UNHCR. (2024). Estudio sobre el desplazamiento forzado de docentes e impacto de la violencia generalizada en centros educativos de Honduras. Available online at: https://data.unhcr.org/en/documents/details/117891 (Accesed June 13, 2025).

[B7] Creative Associates International (2016). Proyecto para reducir factores de riesgo de violencia en familias de alto riesgo en Honduras. Available online at: https://www.creativeassociatesinternational.com/es/story/project-to-reduce-risk-factors-for-violence-among-high-risk-families-in-honduras/ (Accesed Janury 13, 2016).

[B8] DAI Global (2023). Honduras—Asegurando la Educación (Securing Education). Available online at: https://www.dai.com/our-work/projects/honduras-securing-education (Accesed November 11, 2025).

[B9] Elvevåg B. (2025). A voice for the voiceless: a global mental health perspective of the impact of the USAID freeze. Psychiatry Res. 351:116614. doi: 10.1016/j.psychres.2025.116614 40651194

[B10] Evans D. K. Hares S. Smarrelli G. Wu D. (2025). When the data you have aren't the data you need: the availability of school-related violence data in low- and middle-income countries. World Dev. 188:106919. doi: 10.1016/j.worlddev.2025.106919

[B11] Fleckman J. M. Tokarz S. Claire Craig-Kuhn M. Wallace M. E. Theall K. P. (2022). Neighborhood matters: neighborhood violence, collective efficacy, and social emotional development in early childhood. Child. Youth Serv. Rev. 143:106700. doi: 10.1016/j.childyouth.2022.106700

[B12] Fry D. Fang X. Elliott S. Casey T. Zheng X. Li J. . (2018). The relationships between violence in childhood and educational outcomes: a global systematic review and meta-analysis. Child Abuse Negl. 75, 6–28. doi: 10.1016/j.chiabu.2017.06.021 28711191

[B13] Hardaway C. R. McLoyd V. C. Wood D. (2012). Exposure to violence and socioemotional adjustment in low-income youth: an examination of protective factors. Am. J. Community Psychol. 49, 112–126. doi: 10.1007/s10464-011-9440-3 21607826 PMC4071142

[B14] IDLO and INAMI (2021). Niñas, niños y adolescentes en conflicto con la ley penal: *Un estudio cualitativo sobre factores de riesgo y alertas tempranas*. Tegucigalpa: IDLO and INAMI.

[B15] IUDPAS (2024). Boletí*n Nacional 2023*. Available online at: https://iudpas.unah.edu.hn/areas/observatorio-de-la-violencia/boletines-del-observatorio-2/boletines-nacionales/ (Accessed June 18, 2025).

[B16] Koposov R. Isaksson J. Vermeiren R. Schwab-Stone M. Stickley A. Ruchkin V. (2021). Community violence exposure and school functioning in youth: cross-country and gender perspectives. Front. Public Health 9:692402. doi: 10.3389/fpubh.2021.692402 34386472 PMC8353073

[B17] Koslouski J. B. Stark K. Chafouleas S. M. (2023). Understanding and responding to the effects of trauma in the classroom: a primer for educators. Soc. Emot. Learn. Res. Pract. Policy 1:100004. doi: 10.1016/j.sel.2023.100004

[B18] Landa-Blanco M. Echenique Y. Cruz-Quintana F. Fernández-Alcántara M. Pérez Marfil M. N. Navarro E. (2025). Understanding adult attitudes toward corporal punishment in Honduras: the role of sex, gender roles, education, childhood experiences, and psychopathy. J. Interpers. Violence 8:8862605251336785. doi: 10.1177/08862605251336785 40336492

[B19] Landa-Blanco M. Vásquez G. Portillo G. Sproviero F. Echenique Y. (2024). The impact of adverse childhood experiences on mental health, sexual risk behaviors, and alcohol consumption in adulthood. Front. Psychiatry 15:1352824. doi: 10.3389/fpsyt.2024.1352824 38659462 PMC11039929

[B20] McGrath A. (2024). Teachers and students navigating urban violence in Honduras: a view from a school on the margins of El Progreso. Eur. Rev. Latin Am. Caribb. Stud. 118, 23–41. doi: 10.32992/erlacs.11140

[B21] Mrug S. Madan A. Windle M. (2016). Emotional desensitization to violence contributes to adolescents' violent behavior. J. Abnorm. Child Psychol. 44, 75–86. doi: 10.1007/s10802-015-9986-x 25684447 PMC4539292

[B22] Mrug S. Windle M. (2010). Prospective effects of violence exposure across multiple contexts on early adolescents' internalizing and externalizing problems. J. Child Psychol. Psychiatry 51, 953–961. doi: 10.1111/j.1469-7610.2010.02222.x 20331489 PMC3857691

[B23] Pedersen M. L. Holen S. Lydersen S. Martinsen K. Neumer S. P. Adolfsen F. . (2019). School functioning and internalizing problems in young schoolchildren. BMC Psychol. 7:88. doi: 10.1186/s40359-019-0365-1 31870462 PMC6929288

[B24] Rankine J. Fuhrman B. Copperman E. Miller E. Culyba A. (2022). School absenteeism among middle school students with high exposure to violence. Acad. Pediatr. 22, 1300–1308. doi: 10.1016/j.acap.2022.03.012 35342032 PMC9509495

[B25] Suazo M. L. Cruz K. A. (2022). Violencia contra la niñez y la adolescencia en Honduras. Rev. Mex. Sociol. 84, 653–683. doi: 10.22201/iis.01882503p.2022.3.60321

[B26] World Health Organization (2019). School-Based Violence Prevention: A Practical Handbook. Available online at: https://www.unicef.org/media/58081/file/UNICEF-WHO-UNESCO-handbook-school-based-violence.pdf (Accessed June 20 2025).

